# Protective effect of the combinations of glycyrrhizic, ferulic and cinnamic acid pretreatment on myocardial ischemia-reperfusion injury in rats

**DOI:** 10.3892/etm.2014.2134

**Published:** 2014-12-16

**Authors:** YUQIN GAO, JIPING HAO, HONGKAO ZHANG, GUOQIANG QIAN, RENWANG JIANG, JING HU, JIANING WANG, ZHANG LEI, GUOPING ZHAO

**Affiliations:** 1 Department of Cardiology, Ninth Affiliated Hospital of the Medical College of Xi’an Jiaotong University and Railway Central Hospital of Xi’an, Xi’an, Shanxi 710054, P.R. China; 2 College of Medicine, Jinan University, Guangzhou, Guangdong 510632, P.R. China; 3 Department of Nursing Science, Huanghuai University, Zhumadian, Henan 463000, P.R. China; 4 College of Pharmacy, Jinan University, Guangzhou, Guangdong 510632, P.R. China; 5 Department of Cardiology, Renmin Hospital, Hubei University of Medicine, Shiyan, Hubei 442000, P.R. China

**Keywords:** ischemia-reperfusion cocktail, ferulic acid, cinnamic acid, glycyrrhizic acid

## Abstract

The aim of this study was to find an effective drug cocktail pretreatment to protect myocardial tissue of the heart from ischemia-reperfusion (I/R) injury. The mechanisms underlying the effects of the drug cocktail were subsequently explored in order to expand the application of Dang-gui-si-ni-tang (DGSN), a Traditional Chinese Medicine. The active components of DGSN in the serum following oral administration were investigated using high-performance liquid chromatography. The activity of superoxide dismutase (SOD) and malondialdehyde (MDA) levels were then analyzed to show the effect of the active components in the treatment of myocardial I/R injury. An *L*16 (4^4^) orthogonal experiment was utilized to determine the most effective cocktail mix and the mechanism underlying the effect of this mix on myocardial I/R injury was investigated. It was observed that FCG, a mixture of glycyrrhizic (50 mg/kg), cinnamic (200 mg/kg) and ferulic (300 mg/kg) acid, was the optimal drug cocktail present in DGSN. This was absorbed into the blood following oral administration and was shown to decrease MDA levels and increase the activity of SOD. In conclusion, the findings suggest that FCG, a combination of active ingredients in the DGSN decoction, can be absorbed into the blood and protect the myocardium from I/R injury.

## Introduction

Ischemic heart disease is one of the most common causes of mortality in the world (1). Following acute coronary occlusion with the threat of myocardial infarction, the current cardiological protocol for acute myocardial infarction is rapid reperfusion. Although reperfusion is required to salvage ischemic tissue, it is associated with cellular damage due to the activation of deleterious signaling cascades (2). A number of studies have shown that ischemic preconditioning is an innate protective strategy that markedly reduces ischemia-reperfusion (I/R) injury (3–5). This, however, is not acceptable as a clinical tool due to practical difficulties associated with the local induction of cardiac ischemia, ethical reasons and the fact that the index ischemic episode is often unpredictable. Therefore, it is necessary to search for a novel approach, one that is more suitable for the clinical scenario.

Pharmacological preconditioning can simulate ischemic preconditioning and markedly reduce injury from I/R. Furthermore, this method is associated with an easy execution. To date, numerous drugs that can prevent the myocardium from I/R injury (6–8) are available.

Dang-gui-si-ni-tang (DGSN) decoction is a classical formula in Traditional Chinese Medicine that originated from a medical textbook known as the ‘Treatise on Cold-Induced Febrile Diseases’, which dates back to 200 C.E. DGSN is treatment for coronary heart disease. Studies have indicated that glycyrrhizic, ferulic and cinnamic acids are the active components in the DGSN decoction (9–11). In addition, the majority of these studies have reported that glycyrrhizic, cinnamic and ferulic acids have a protective effect against I/R injury. The protective mechanism of glycyrrhizic acid on I/R injury was found to be associated with its antioxidant (12–14), anti-inflammatory (15) and anti-apoptosis effects (16), as well as its inhibition of lipid peroxide (17). Cinnamic acid exerts cytoprotection by acting as an antioxidant and anti-inflammatory agent (18–20). Ferulic acid can also inhibit oxidative stress, inflammation (21) and cell apoptosis (22) and modulate mitochondrial function (23). These mechanisms are associated with the alleviation of I/R injury. Considering that glycyrrhizic, cinnamic and ferulic acids are major components of the DGSN decoction, which is used to treat coronary heart diseases (9–11), we hypothesized that different combinations of the active ingredients in the DGSN decoction may also have a similar therapeutic effect.

The aims of this study were fourfold. Firstly, we aimed to verify whether glycyrrhizic, cinnamic and ferulic acids could be absorbed into the serum of rats following oral administration of the DGSN decoction. We then aimed to investigate the effects of pretreament with glycyrrhizic, cinnamic and ferulic acids and peoniflorin on superoxide dismutase (SOD) activity and malondialdehyde (MDA) levels in the myocardium of a rat model of I/R injury, and to utilize an *L*16 (4^4^) orthogonal experiment to find the optimal active combination. Thirdly, we aimed to investigate the protective effects of the optimal active combination pretreatment against myocardial I/R injury in rats, and, finally, to investigate whether the protective effects were associated with tumor necrosis factor-α (TNFα), interleukin (IL)-1β, IL-6 and the nuclear factor-κB (NF-κB)p65 and peroxisome proliferator-activated α (PPARα) signaling pathways.

## Materials and methods

### Materials

Male Sprague Dawley (SD) rats weighing 280–400 g were purchased from the Guangdong Experimental Animal Center (License no. SCXK 2008-0002; Guangzhou, China). The animals were maintained in individual cages at room temperature under light-controlled conditions. The rats were provided with food and water *ad libitum*. All animal procedures were in accordance with the Regulations of Experimental Animal Administration issued by the State Committee of Science and Technology of the People’s Republic of China on November 14, 1988. Glycyrrhizic acid (purity >98%), ferulic acid (purity >99%), peoniflorin (purity >98%) and cinnamic acid (purity >99%) were purchased from Nanjing Zelang Medical Technology Co., Ltd. (Nanjing, China). The following primary antibodies, which were obtained from Santa Cruz Biotechnology, Inc. (Santa Cruz, CA, USA) unless stated, were used: Anti-phosphorylated (phospho)-Akt1/2 [serine (Ser)473] (cat. no. 9271; Cell Signaling Technology, Inc. Danvers, MA, USA), anti-inhibitory(I)-κBα (cat. no. SC-101712), anti-phospho-I-κBα (Ser32/36) (cat. no. SC-101713), anti-NF-κBp65 (cat. no. SC-7151), anti-GAPDH (cat. no. SC-25778) and anti-PPARα (cat. no. SC-9000). Horseradish peroxidase-conjugated goat anti-rabbit immunoglobulin G (IgG; Heavy and Light chain) was purchased from Beijing Biosynthesis Biotechnology Co., Ltd. (Beijing, China). SOD, MDA, TNFα ELISA, IL-1β ELISA and intercellular adhesion molecule-1 (ICAM-1) ELISA test kits, as well as pentobarbital and 2,3,5-triphenyltetrazolium chloride (TTC), were purchased from Nanjing Jiancheng Technology Co., Ltd. (Nanjing, China). Enhanced chemiluminescence diagnostic, bicinchoninic acid (BCA) protein assay and nucleoprotein and plasmosin extraction kits were purchased from Nanjing KeyGen Biotech. Co., Ltd. (Nanjing, China).

### Preparation of the DGSN decoction

The ratio of *Angelica sinensis*, Radix Paeonia, Ramulus Cinnamomi, Herba Asari Mandshurici, Radix Glycyrrhizae, Medulla Tetrapanacis and Fructus Jujubae in DGSN was 4:3:3:1:2:2:2. The DGSN was purchased from the First Affiliated Hospital of Guangzhou University of Traditional Chinese Medicine (Guangzhou, China). The DGSN was boiled twice in distilled water (1:12, w/v) for 30 min. The blended supernatants were then condensed to a concentration of 1 g crude drug/ml. Liquid extraction (500 μl) was mixed with 2 ml blank rat serum. The mixed liquid was acidified with 20 μl acetic acid and extracted with 8 ml n-butanol. Subsequent to centrifugation at 400 × g for 20 min at 4–6°C, the organic phase was transferred into an empty tube and evaporated to dryness under nitrogen at 40°C. The residue was dissolved in 1 ml methanol and filtered (0.22 μm; Millipore, Billerica, MA, USA), and an aliquot (20 μl) was then injected into the high-performance liquid chromatography (HPLC) system.

### Preparation of reference compounds and HPLC analysis

The purity of the glycyrrhizic, cinnamic and ferulic acids and peoniflorin was >98%. The chromatographic analysis was performed on an Agilent 1200 system (Agilent Technologies, Palo Alto, CA, USA), which was composed of a quaternary gradient pump, an auto sampler, a Cosmosil C-18 column (5 μM particle, 250×4.6 mm) and an ultraviolet detector. The mobile phase was acetonitrile-0.5% aqueous acetic acid. The analysis was performed at a flow-rate of 1.0 ml/min with detections at 320, 230, 275 and 254 nm. The gradient solvent system is shown in Table I.

All reference compounds were dissolved in dimethyl sulfoxide to afford 50 mmol/l stock solutions separately. The concentration of mixed standards were as follows (where G, F, P and C stand for glycyrrhizic acid, ferulic acid, peoniflorin and cinnamic acid, respectively): Mixed standard 1 (G: 4×10^−8^ mol/l, F: 2×10^−8^ mol/l, P: 2×10^−8^ mol/l and C: 2×10^−8^ mol/l); Mixed standard 2 (G: 8×10^−8^ mol/l, F: 4×10^−8^ mol/l, P: 4×10^−8^ mol/l and C: 4×10^−5^ mol/l); Mixed standard 3 (G: 1.6×10^−7^ mol/l, F: 8×10^−8^ mol/l, P: 8×10^−8^ mol/l and C: 8×10^−8^ mol/l); Mixed standard 4 (G: 3.2×10^−7^ mol/l, F: 1.6×10^−7^ mol/l, P: 1.6×10^−7^ mol/l and C: 1.6×10^−7^ mol/l); and Mixed standard 5 (G: 6.4×10^−7^ mol/l, F: 3.2×10^−7^ mol/l, P: 3.2×10^�7^ mol/l and C: 3.2×10^−7^ mol/l). The mixed standards were mixed separately with 2 ml blank rat serum, respectively; the mixed liquid was acidified with 20 μl acetic acid and extracted with 8 ml n-butyl alcohol. Following centrifugation at 400 × g for 20 min at 4–6°C, the organic phase was transferred into an empty tube and evaporated to dryness under nitrogen at 40°C. The residue was dissolved in 1 ml methanol and filtered (0.22 dm; Millipore). An aliquot (20 μl) was then injected into the HPLC system.

### Serum sample preparation

The 35 male rats were housed in an environmentally controlled room and divided randomly into seven groups (five rats in each group). The seven groups represented different time periods, i.e. 30, 60, 90, 120, 180, 360 and 540 min). The DGSN decoction was orally administered to the rats at a dosage of 20 g/kg. The abdominal cervical artery was then punctured under pentobarbital sodium anesthesia and the blood (5 ml) was collected. Each rat yielded 2 ml serum. The serum samples were processed according to the same procedure as the standard samples. An aliquot (20 μl) was injected into the HPLC system.

### Experimental protocols

In the first set of experiments, it was investigated whether the DGSN decoction contained the ferulic acid, peoniflorin, cinnamic acid and glycyrrhizic acid (FPCG) combinations and, if so, whether these active components were absorbed into the blood following oral administration of the DGSN decoction. Animal experiments were then performed based on an *L*16 (4^4^) orthogonal design (24–26), setting four factors with four different levels (Table II).

SD rats were randomly divided into the above 16 groups (n=6 per group). Prior to surgery, the rats were administrated different doses of the GFCP combinations for 5 days (once a day, five doses in total). The last dose was administered 30 min prior to the surgery.

In the third set of experiments, the SD rats were randomly divided into three groups (n=10 per group). The first group underwent the same procedure as groups II and III, except the suture was passed under the coronary artery without ligation (sham group). In group II, the rats underwent 30 min ischemia followed by 2 h reperfusion. In group III, the rats were administered the FCG combination (glycyrrhizic acid, 50 mg/kg; ferulic acid, 300 mg/kg and cinnamic acid, 200 mg/kg) for five days prior to I/R. At the end of the experiments, 4–5 ml blood was obtained. The serum was separated from the blood cells by centrifugation and stored at −80°C. For further analysis, the hearts were prepared for infarct size measurement or the tissue was quickly frozen and stored at −80°C.

### Surgical preparations

The surgical protocol was performed according to methods described previously (27). Briefly, rats were anesthetized with pentobarbital (50 mg/kg, intraperitoneal), intubated and ventilated with mechanical ventilation (tidal volume, 30 ml/kg; 70 strokes per min). The rats were placed on heating plates to maintain core temperature within the normal range (37.0–37.6°C) and the left femoral vein was cannulated to inject the drugs. With the fourth intercostal space opened, the heart was exteriorized and the pericardium was cut. The left anterior descending coronary artery was ligated between the left atrium and the pulmonary outflow tract using a 6-0 silk suture. Successful ligation was verified by regional cyanosis of the myocardial surface and ischemic ST-segment changes in the electrocardiogram. The heart was subsequently replaced in the thoracic cavity, the thoracic cavity was drained of remaining air (to avoid pneumothorax) and the chest was immediately closed. Following occlusion for 30 min, the left anterior descending coronary artery was opened to permit reperfusion for 2 h.

### Measurement of area at risk (AAR) and infarct size (IS)

Myocardial IS and AAR were determined as described previously (27). Briefly, at the end of reperfusion period, the rat hearts were removed and the aortas were quickly cannulated. Once the coronary artery ligature was tied, the hearts were perfused with Evan’s blue at a constant pressure (80 mmHg). The atria and the right ventricle were removed, and the left ventricle (LV), including the septum, was cut into 2- to 3-mm slices from the apex to the base. The perfused myocardium was stained blue, whereas the AAR remained unstained. The AAR was determined as the percentage of the ischemic myocardial mass against the LV myocardial mass. The unstained myocardium was incubated for 30 min at 37°C in TTC (1% in 0.1 mol/l phosphate buffer, pH 7.4). The noninfarcted myocardium was deep red, in contrast to the pale white of the infarcted myocardium. The IS was expressed as the percentage of the infarcted myocardial mass against the ischemic myocardial mass.

### Biochemical parameters

The serum obtained from the rats was used for the measurement of MB-isoenzyme of creatine kinase (CK-MB), TNFα, IL-1β, IL-6 and ICAM-1 levels. CK-MB level was determined following the homogenization of the rat myocardium in lysis buffer from BioTeke Corporation (Beijing, China). TNFα, IL-1β, IL-6, ICAM-1 aand MDA levels, and SOD activity were determined following the homogenization of the rat myocardium in lysis buffer from Nanjing Jiancheng Technology Co., Ltd. In addition, the protein content of all tissues was determined using the BCA method.

### Electron microscopy

For the ultrastructural morphological study, the samples were fixed with 2.5% glutaraldehyde solution, and then dehydrated in a graded series of ethanol, 1% O_5_O_4_, phosphate-buffered saline and acetone, and embedded in Epon812 embedding medium. The ultrathin sections were prepared on a Reichert-Jung Ultracut E ultramicrotome (Leica Corporation, Shanghai, China), picked up on copper grids and stained. Specimens were observed under a JEM-100CX electron microscope (JEOL Japan Electronics Co., Ltd. Tokyo, Japan).

### Histological examination

Myocardial tissues of SD rats were fixed in 10% neutral formaldehyde for 24 h, and then each sample was dehydrated with segments embedded in paraffin and cut into 5-μm thick sections for staining with hematoxylin and eosin (H&E).

### Western blot analysis

Rat myocardium was homogenized in lysis buffer (50 mm Tris pH 7.4, 150 mm NaCl, 1% Triton X-100, 1% sodium deoxycholate, 0.1% SDS, EDTA and protease inhibitor cocktail) and centrifuged at 3,000 × g for 10 min. Protein concentration was determined using a BCA protein assay kit according to the manufacturer’s instructions (Nanjing KeyGen Biotech. Co., Ltd.). Equal amounts of protein (100 μg/sample) were electrophoresed by SDS-PAGE and transferred onto a polyvinylidene difluoride membrane. The membrane was blocked with 5% non-fat milk in 1X Tris buffered saline and 0.1% Tween 20 at room temperature for 3 h. The membrane was then incubated overnight at 4°C with the appropriate primary antibody [anti-phospho-I-κBα (Ser32/36), anti-I-κBα, anti-NF-κBp65, anti-PPARα, anti-GAPDH or anti-phospho-Akt1/2] diluted in Tris buffered saline/Tween 20 (Tris buffered saline, 0.1% Tween 20). The working concentrations of the anti-phospho-I-κBα (Ser32/36), anti-I-κBα, anti-NF-κBp65, anti-PPARα, anti-pAkt1/2 and anti-GAPDH antibodies were 1:500, 1:1,000, 1:1,000, 1:1,000, 1:500 and 1:2,000, respectively. Following incubation with peroxidase-conjugated goat anti-rabbit IgG secondary antibodies for 1 h, the blots were developed with chemiluminescence reagent and exposed to X-ray film. Nuclear protein was then extracted using an extraction kit according to the manufacturer’s instructions (Nanjing KeyGen Biotech. Co., Ltd.). Band intensities were quantified using a densitometer analysis system (Quantity One^®^, Bio-Rad, Hercules, CA, USA).

### Statistical analysis

Data are presented as the mean ± standard deviation. Statistical analysis for the experimental groups was performed using SPSS for Windows version 13.0 (SPSS, Inc., Chicago, IL, USA). Differences among groups were compared with one-way analysis of variance (ANOVA) followed by the Dunnett’s test. Differences were considered statistically significant when P<0.05. The variance analysis was applied in the orthogonal experiment.

## Results

### Detection of serum components by HPLC following oral administration of DGSN

**Figure 1:**
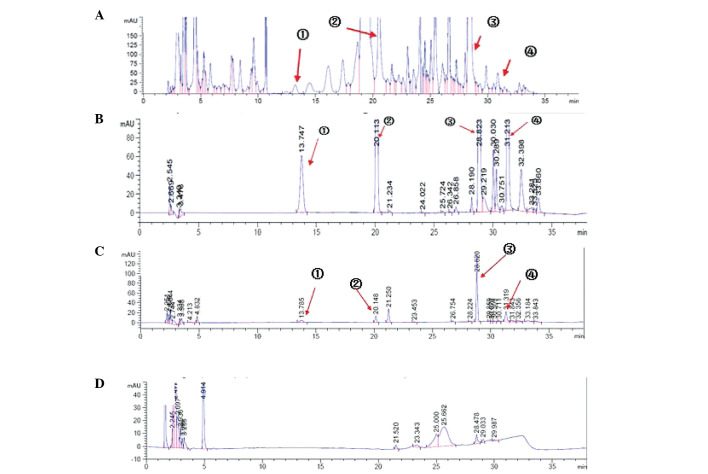
High-performance liquid chromatography chromatograms of (A) Dang-gui-ni-tang decoction extracted by n-butanol; (B) the standard sample; (C) the serum sample; and (D) the blank serum. The peaks marked as ➀ ➁ ➂ and ➃ are peoniflorin and ferulic, cinnamic and glycyrrhizic acids, respectively. The detection wavelength was 275 nm.

The results (Fig. 1A–D) showed the DGSN decoction contained FPCG detected in serum of rats following oral administration of the DGSN decoction by HPLC analysis.

### Orthogonal-design experiment results

In the orthogonal experiments (Tables III–VI), SOD activity was assessed using the xanthine oxidase method and MDA levels were measured using the thiobarbituric acid-reactive-substances assay. Through ANOVA and the orthogonal experiment intuitionistic approach, it was found that glycyrrhizic acid was the most effective component at increasing SOD activity in the rat myocardium following I/R; ferulic acid, peoniflorin and cinnamic acid were second-most, third-most and least effective, respectively. The optimal drug combination was glycyrrhizic acid (50 mg/kg), cinnamic acid (0 mg/kg), ferulic acid (0 mg/kg) and peoniflorin (0 mg/kg). In addition, it was found that cinnamic acid was the most effective component at reducing MDA levels in the rat myocardium following I/R; ferulic acid, peoniflorin and glycyrrhizic acid were second-most, third-most and least effective, respectively. The optimal drug combination was cinnamic acid (200 mg/kg), ferulic acid (300 mg/kg), peoniflorin (0 mg/kg) and glycyrrhizic acid (0 mg/kg). Taking both sets of results into consideration, the combination of glycyrrhizic acid (50 mg/kg), cinnamic acid (200 mg/kg) and ferulic acid (300 mg/kg) (FCG) was regarded as optimal. This not only lowered the content of MDA, but also increased the activity of SOD.

### Effect of FCG pretreatment on biochemical parameters induced by I/R injury

TNFα, IL-1β, IL-6 and ICAM-1 levels were detected by ELISA assay, and the presence of CK-MB was revealed by enzyme rate assay. As shown in Tables VII and VIII, the levels of TNFα, IL-1β, IL-6, ICAM-1 and CK-MB were increased significantly in the myocardium of rats with I/R injury as compared with those in the myocardium of sham-operated rats. However, administering FCG pretreatment for five days before the I/R injury significantly decreased the serum levels of TNFα, IL-1β, IL-6, ICAM-1 and CK-MB as compared with the levels in the I/R group without pretreatment.

### Effect of FCG pretreatment on myocardial IS and AAR

**Figure 2:**
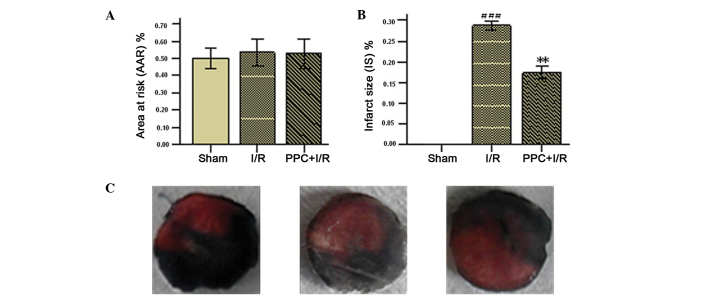
Effects of FCG on (A) myocardial AAR and (B) IS. (C) Images of the myocardial AAR and infarct area in the sham, I/R and PPC+I/R groups (left, center and right, respectively). The area of infarct is white, the myocardial AAR is deep red and the nonischemic area is blue. Data are presented as the mean ± standard deviation; n=6. ^**^P<0.01 vs. I/R; ^###^P<0.001 vs. sham, n=6. Sham, sham-operated animal without ligation; I/R, 30 min ischemia followed by 2 h reperfusion; PPC+I/R, administration of FCG for 5 days prior to the induction of myocardial ischemia. FCG, ferulic acid (300 mg/kg), cinnamic acid (200 mg/kg) and glycyrrhizic acid (50 mg/kg); AAR, area at risk; IS, infarct size.

The AAR and IS are a percentage of the LV and AAR weights, respectively. AAR and IS were used to assess the efficacy of the FCG combination in the protection of the rat myocardium following I/R injury. Significant differences were not apparent among the three groups with regard to the AAR. The values for the IS were 0.17±0.01% in the FCG pretreatment group and 0.29±0.08% in the I/R group. Significant differences in IS were observed between the FCG pretreatment group (PPC plus I/R) and the I/R group (Fig. 2A–C).

### Effect of FCG pretreatment on histological changes induced by I/R injury

**Figure 3:**
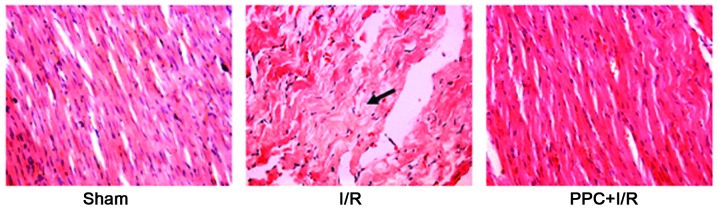
Morphology of rat myocardium (hematoxylin and eosin staining; magnification, ×200). Sham, sham-operated animal without ligation; I/R, 30 min ischemia followed by 2 h reperfusion; PPC+I/R, administration of FCG for 5 days prior to the induction of myocardial ischemia. The arrow indicates muscle fiber disarranging and clear hydropic degeneration. FCG, ferulic acid (300 mg/kg), cinnamic acid (200 mg/kg) and glycyrrhizic acid (50 mg/kg).

The histomorphology of the cardiac muscle of the LV of the rats was observed using H&E staining. The following changes were found in the I/R group: Muscle fiber disarrangement, clear hydropic degeneration, cell dropsy, dark nuclear staining, vascular bleeding, inflammatory cell infiltration and myocardial fiber atrophy. These morphological changes were alleviated by FCG pretreatment (Fig. 3).

### Effect of FCG pretreatment on ultrastructural changes induced by I/R injury

**Figure 4:**
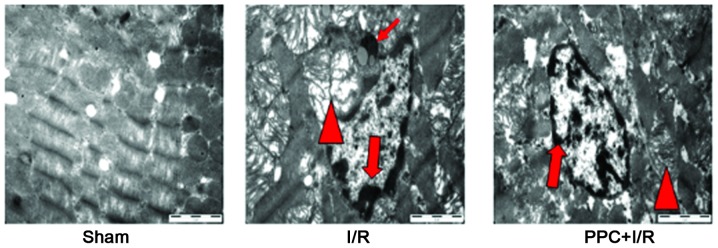
Electron microscopic examination of rat myocardium (magnification, ×15,000). Sham, sham-operated animal without ligation; I/R, 30 min ischemia followed by 2 h reperfusion; PPC+I/R, administration of FCG for 5 days prior to the induction of myocardial ischemia. Thin arrows indicate apoptotic bodies, Δ indicates swelling mitochondria, large arrows indicate chromatin condensation and aggregation. FCG, ferulic acid (300 mg/kg), cinnamic acid (200 mg/kg) and glycyrrhizic acid (50 mg/kg).

The ultrastructural changes in the cardiac muscle of the LV of the rats were observed by transmission electron microscopy (Fig. 4). These changes included cell swelling, mitochondrial swelling, cristae disorganization, myofibril shrinkage and lysis, chromatin condensation and aggregation at the periphery of the nucleus and nuclear fragmentation. Apoptotic bodies were also observed in the I/R group. The aforementioned ultrastructural changes were alleviated in the PPC plus I/R group as compared with the I/R group.

### Effect of FCG pretreatment on the expression of I-κBα, NF-κB, p65, PPARα and pAkt1/2 induced by I/R injury

**Figure 5:**
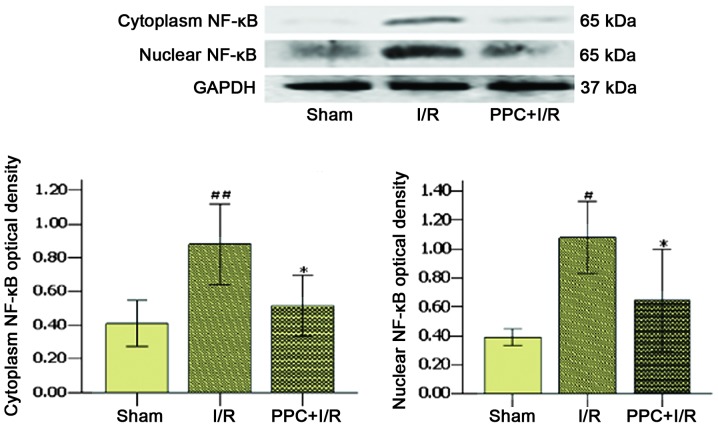
Effect of FCG pretreatment on cytoplasmic and nuclear NF-κBp65 expression in different groups. Data are presented as the mean ± standard deviation; n=6. ^*^P<0.05 vs. I/R; ^##^P<0.001 and ^#^P<0.05 vs. sham. Sham, sham-operated animal without ligation; I/R, 30 min ischemia followed by 2 h reperfusion; PPC+I/R, administration of FCG for 5 days prior to the induction of myocardial ischemia. FCG, ferulic acid (300 mg/kg), cinnamic acid (200 mg/kg) and glycyrrhizic acid (50 mg/kg); NF-κB, nuclear factor-κB.

**Figure 6:**
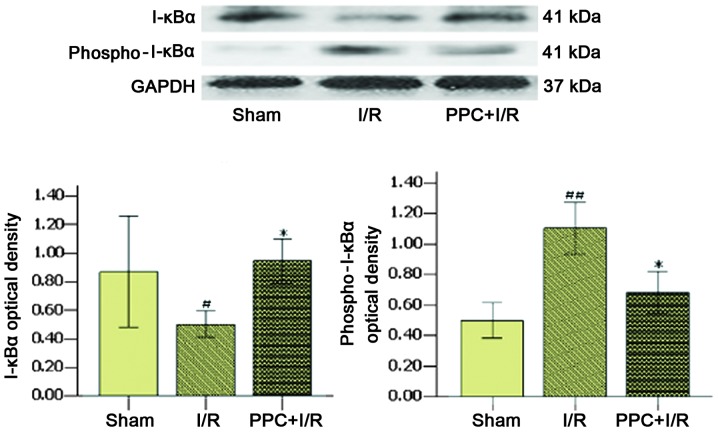
Effect of FCG pretreatment on cytoplasmic I-κBα and phospho-I-κBα expression in different groups. Data are presented as the mean ± standard deviation; n=6. ^*^P<0.05 vs. I/R; ^##^P<0.001 and ^#^P<0.05 vs. sham. Sham, sham-operated animal without ligation; I/R, 30 min ischemia followed by 2 h reperfusion; PPC+I/R, administration of FCG for 5 days prior to the induction of myocardial ischemia. FCG, ferulic acid (300 mg/kg), cinnamic acid (200 mg/kg) and glycyrrhizic acid (50 mg/kg); I-κBα, inhibitory-κBα; phospho-, phosphorylated-.

**Figure 7:**
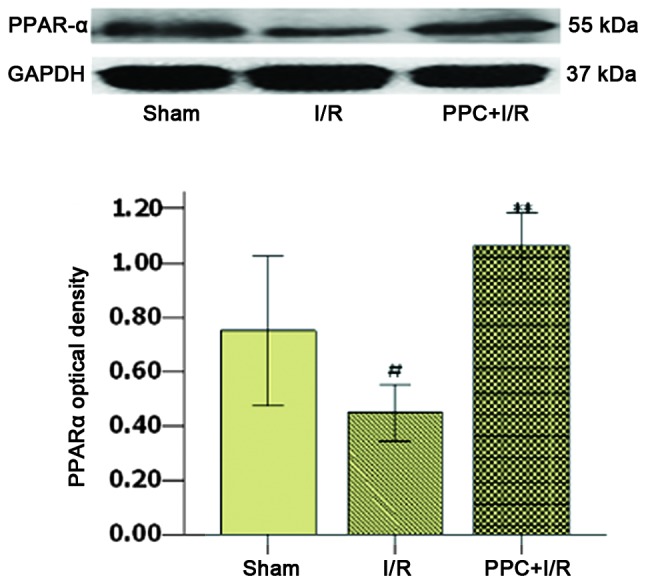
Effect of FCG pretreatment on PPARα protein expression in different groups. Data are presented as the mean ± standard deviation; n=6. ^**^P<0.05 vs. I/R; ^#^P<0.05 vs. sham. Sham, sham-operated animal without ligation; I/R, 30 min ischemia followed by 2 h reperfusion; PPC+I/R, administration of FCG for 5 days prior to the induction of myocardial ischemia. FCG, ferulic acid (300 mg/kg), cinnamic acid (200 mg/kg) and glycyrrhizic acid (50 mg/kg); PPARα, peroxisome proliferator-activated receptor α.

**Figure 8:**
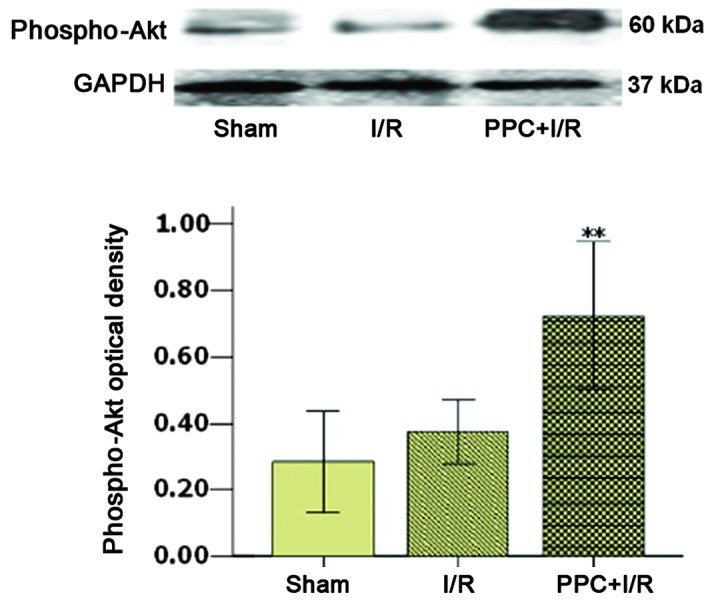
Effect of FCG pretreatment on phospho-Akt protein expression in different groups. Data are presented as the mean ± standard deviation; n=6. ^**^P<0.05 vs. I/R. Sham, sham-operated animal without ligation; I/R, 30 min ischemia followed by 2 h reperfusion; PPC+I/R, administration of FCG for 5 days prior to the induction of myocardial ischemia. FCG, ferulic acid (300 mg/kg), cinnamic acid (200 mg/kg) and glycyrrhizic acid (50 mg/kg); phospho-, phosphorylated-.

The effects of pharmacological pretreatment with FCG on nuclear NF-κBp65, cytoplasmic NF-κBp65, IκBα, phospho-IκBα, PPARα and phospho-Akt proteins in the myocardium of rats were detected by western blot analysis (Figs. 5–8). Compared with the sham group, the expression levels of NF-κBp65 and phospho-I-κB were significantly increased in the I/R group, whereas the expression levels of I-κB and PPARα were decreased. The levels of phospho-Akt were increased in the I/R group, although the difference was not significant. However, compared with the I/R group, the expression of NF-κBp65 and phospho-I-κB was significantly decreased, whereas the expression of I-κB, PPARα and phospho-Akt was increased in the PPC plus I/R group.

## Discussion

The results of the present study showed that, while a number of chemical constituents were contained in DGSN decoction, a Traditional Chinese Medicine (Fig. 1A), <10 types of constituent could be absorbed into the blood (Fig. 1C) following oral administration of the DGSN decoction. Only the components that can be absorbed into the blood may play a role in pharmacology. Therefore, the objective of our study was to investigate which components could be absorbed following oral administration of DGSN decoction, and to then find the optimal drug cocktail composition from the absorbable components, in order to treat I/R injury of the rat myocardium through orthogonal experiments. The mechanism of the optimal cocktail composition was then further explored.

DGSN effectively treats coronary heart diseases. Cinnamic, glycyrrhizic and ferulic acids, and peoniflorin can be detected in DGSN decoction (9–11), and in the serum of rats following the oral administration of DGSN decoction (Fig. 1A–D). A number of studies have reported that glycyrrhizic (28), ferulic (29,30) and cinnamic (31,32) acids can alleviate damage following I/R injury, respectively. Therefore, it may be hypothesized that the optimal drug combination for treating rat myocardial I/R injury may be derived from the components of DGSN.

In multifactor experiments, an orthogonal experiment design enables the hierarchical status of the factors and the interaction among the factors to be established, which facilitates the determination of the ideal combination of multiple factors with as few sampling experiments as possible. In pharmacodynamic studies of multiple factors, the optimal drug combinations can be revealed by orthogonal experiment design. The *L*16 (4^4^) orthogonal design was selected in the present study to find an optimal cocktail drug combination from the components absorbed following the oral administration of DGSN decoction. The strength of effect as well as correlations among components in the cocktail drug combinations were detected. SOD activity and MDA levels represent an evaluation index to measure the effectiveness among these components and cocktail drug combinations, since these indexes can respond to the damage degrees of oxidative stress and inflammation. Reperfusion is essential for myocardial tissue survival. However, its effect on ischemic myocardium is two-fold, as it can trigger oxidative tissue damage and inflammation (23,27,33,34). Furthermore, the inflammatory response and oxidative damage are important causes of myocardial I/R injury (35). The MDA content can respond to the degree of lipid peroxidation, and the modulation of antioxidant enzymes, such as SOD and catalase, can protect against oxidative cardiac disorders (36,37).

In the present study, the *L*16 (4^4^) orthogonal experiment intuitionistic analytical approach was used to find the most effective component at reducing MDA levels in the rat myocardium following I/R. The most effective component was cinnamic acid, followed by ferulic acid, peoniflorin and glycyrrhizic acid. However, ANOVA showed that only cinnamic acid and ferulic acid were capable of significantly decreasing the content of MDA (Table V). In addition, it was found that the most effective component at increasing SOD activity in the rat myocardium following I/R was glycyrrhizic acid, followed by ferulic acid, peoniflorin and cinnamic acid. However, ANOVA showed that only glycyrrhizic acid significantly increased the activity of SOD (Table III). Summarizing the results of the SOD activity and MDA level analysis, FCG was revealed to be the optimal drug combination, consisting of glycyrrhizic (50 mg/kg), cinnamic (200 mg/kg) and ferulic (300 mg/kg) acid. This combination not only reduced the content of MDA, but also increased the activity of SOD.

In the present study the second step was to explore the mechanism underlying the effect of FCG on I/R injury. The effects could be observed in a number of aspects, including myocardial IS, myocardial tissue construction, cell ultrastructure, inflammatory and biochemical parameters, signaling pathways of the inflammatory response and oxidative damage. The results showed that FCG could decrease myocardial IS (Fig. 2). The following changes were also revealed: Muscle fiber disarrangement, clear hydropic degeneration, cell dropsy, dark nuclear staining, vascular bleeding and inflammatory cell infiltration. These changes were significantly alleviated by FCG pretreatment compared with the I/R group. In addition, the cardiac muscle ultrastructural changes were observed via transmission electron microscopy (Fig. 4). These included cell swelling, mitochondrial swelling, cristae disorganization, myofibril shrinkage and lysis. Chromatin condensation and aggregation at the periphery of the nucleus and nuclear fragmentation were shown to be significantly alleviated by FCG pretreatment compared with the I/R group.

NF-κB is an important transcriptional regulatory factor and plays an important role in myocardial I/R injury. Furthermore, NF-κB has a close association with the inflammatory response and oxidative damage. I/R injury can cause a rapid phosphorylation of I-κBα and degradation of I-κBα (I-κBα is the inhibitory protein of NF-κB activation). This leads to the activation of NF-κB translocation into the nucleus and the transcription of other downstream inflammatory factors, including TNFα, IL-1β, IL-6 and ICAM-1 (38–40). PPARα is an important regulatory factor of NF-κB (41,42) that, following activation by its ligands, can suppress the activation and nuclear translocation of NF-κB (43), repress the expression of inflammatory factors and increase the expression of SOD (41,44–46). PPARα is closely associated with the Akt signal transduction pathway (47). In the present study, the expression levels of TNFα, IL-1β, IL-6, ICAM-1, NF-κBp65 and phospho-I-κBα were detected to be significantly increased in the I/R group, while the expression levels of I-κBα, PPARα were significantly decreased. The levels of phospho-Akt were increased in the I/R group compared with the sham group, although the difference was not significant. However, compared with the I/R group, the expression levels of TNFα, IL-1β, IL-6 and ICAM-1 significantly decreased with FCG pretreatment. Furthermore, the expression levels of NF-κBp65 and phospho-I-κBα significantly decreased, and the expression of I-κBα, PPARα and phospho-Akt increased. The results of this study suggested that FCG pretreatment protected the myocardium from I/R injury by activating Akt and PPARα, decreasing I-κBα phosphorylation and further repressing NF-κBp65. However, the results did not confirm that the protective effect of FCG pretreatment against I/R injury in the rat myocardium was exerted by the direct activation of Akt. The activation of Akt can facilitate FCG to activate PPARα and further inhibit the phosphorylation of I-κBα to downregulate the expression and activity of NF-κBp65. However, it is not known whether FCG can directly inhibit the expression of NF-κBp65 through the interaction of Akt, PPARα, I-κBα and NF-κB. Further studies are in progress.

In conclusion, we have found and validated that cinnamic acid, glycyrrhizic acid and ferulic acid are present in DGSN decoction, and can be absorbed into the blood of rats following oral administration of DGSN decoction. The optimal drug combination, FCG, is a mixture of glycyrrhizic (50 mg/kg), cinnamic (200 mg/kg) and ferulic (300 mg/kg) acid. FCG was shown to not only lower the content of MDA but also increase the activity of SOD, as demonstrated by the *L*16 (4^4^) orthogonal design. These findings show that FCG may alleviate myocardial I/R injury. It was suggested that FCG acted by significantly decreasing the levels of TNFα, IL-1β, IL-6 and ICAM-1 in the serum. This significantly decreased the myocardial AAR and IS and alleviated the changes in myocardial tissue construction and cell ultrastructure observed by H&E staining and transmission electron microscopy. In addition, FCG significantly increased the expression of I-κBα, PPARα and phospho-Akt, and decreased the expression levels of NF-κBp65 and phospho-I-κBα. Accordingly, the results of the present study indicated that the FCG drug combination protected the myocardium from I/R injury through modulation of the Akt, PPARα and NF-κB pathways.

## Figures and Tables

**Table I:** Program of gradient elution.

Time, min	Φ (acetonitrile), %	*V* (0.5% aqueous acetic acid), %
0–12	15	85
12–30	50	50
30–33	15	85
33–38	15	85

**Table II:** Four levels of the four active components in the orthogonal design.

	Glycyrrhizic acid (mg/kg)	Ferulic acid (mg/kg)	Peoniflorin (mg/kg)	Cinnamic acid (mg/kg)
	
Level	A	B	C	D
1	0	0	0	0
2	25	200	25	100
3	50	300	50	200
4	100	400	100	400

**Table III:** Effect of FPCG on increasing superoxide dismutase activity in the rat myocardium following ischemia-reperfusion.

Source of variation	Mean-square	*F*	P-value	n
Ferulic acid	130.805	1.958	0.155	6
Peoniflorin	22.190	0.332	0.802	6
Cinnamic acid	15.898	0.238	0.869	6
Glycyrrhizic acid	322.778	4.833	0.012	6

Results were assessed by analysis of variance. FPCG, ferulic acid, peoniflorin, cinnamic acid and glycyrrhizic acid combination.

**Table IV:** Effect of FPCG on increasing SOD activity in the rat myocardium following ischemia-reperfusion.

Group	Glycyrrhizic acid (A)	Ferulic acid (B)	Peoniflorin (C)	Cinnamic acid (D)	SOD (U/mg)
1	1	1	1	1	25.63±7.73
2	3	3	1	3	50.01±1.97
3	4	4	1	4	48.21±7.83
4	2	2	1	2	35.54±4.73
5	2	4	3	1	38.85±0.79
6	4	3	2	1	46.89±1.05
7	3	2	4	1	49.96±7.48
8	1	4	4	3	36.23±0.66
9	4	1	4	2	45.12±8.51
10	1	3	3	2	43.30±4.49
11	2	3	4	4	40.71±4.04
12	2	1	2	3	31.86±0.97
13	3	1	3	4	41.26±4.59
14	3	4	2	2	47.21±6.67
15	4	2	3	3	40.21±6.05
16	1	2	2	4	30.85±5.92
K_1_	34.00	35.96	39.85	40.33	-
K_2_	36.74	39.14	39.20	42.79	-
K_3_	47.11	45.23	40.90	39.58	-
K_4_	45.11	42.62	43.00	40.25	-
R_j_	13.10	9.26	3.80	2.54	-

1, 2, 3 and 4 represent no drug, low dose, medium dose and high dose, respectively. Data were analyzed using the orthogonal experiment intuitionistic analytical approach and are presented as the mean ± standard deviation. SOD, superoxide dismutase; FPCG, ferulic acid, peoniflorin, cinnamic acid and glycyrrhizic acid combination; K_x_, mean of a certain factor K at a particular level x; R_j_, range of the column (denoted as J) of a certain factor.

**Table V:** Effect of FPCG on reducing malondialdehyde levels in the rat myocardium following ischemia-reperfusion.

Source of variation	Mean-square	*F*	P-value	n
Ferulic acid	0.167	3.32	0.032	6
Peoniflorin	0.119	2.367	0.090	6
Cinnamic acid	0.231	4.582	0.009	6
Glycyrrhizic acid	0.067	1.337	0.280	6

Results were assessed by analysis of variance. FPCG, ferulic acid, peoniflorin, cinnamic acid and glycyrrhizic acid combination.

**Table VI:** Effect of FPCG on reducing MDA levels in the rat myocardium following ischemia-reperfusion.

Group	Glycyrrhizic acid	Ferulic acid	Peoniflorin	Cinnamic acid	MDA (nmol/mg)
1	1	1	1	1	0.99±0.11
2	3	3	1	3	0.43±0.07
3	4	4	1	4	0.48±0.31
4	2	2	1	2	0.43±0.12
5	2	4	3	1	1.19±0.03
6	4	3	2	1	0.66±0.11
7	3	2	4	1	0.65±0.20
8	1	4	4	3	0.58±0.32
9	4	1	4	2	0.54±0.05
10	1	3	3	2	0.45±0.12
11	2	3	4	4	0.40±0.07
12	2	1	2	3	0.58±0.12
13	3	1	3	4	0.73±0.14
14	3	4	2	2	0.89±0.23
15	4	2	3	3	0.48±0.12
16	1	2	2	4	0.86±0.30
K_1_	0.72	0.71	0.58	0.62	-
K_2_	0.65	0.61	0.75	0.58	-
K_3_	0.67	0.48	0.71	0.52	-
K_4_	0.54	0.78	0.54	0.87	-
R_j_	0.18	0.30	0.21	0.36	-

1, 2, 3 and 4 represent no drug, low dose, medium dose and high dose, respectively. Data were analyzed using the orthogonal experiment intuitionistic analytical approach and are presented as the mean ± standard deviation. MDA, malondialdehyde; FPCG, ferulic acid, peoniflorin, cinnamic acid and glycyrrhizic acid combination.

**Table VII:** Effects of FCG pretreatment on serum TNFα and ICAM-1 levels in the rat myocardium following ischemia-reperfusion.

Group	TNFα (ng/l)	ICAM-1 (ng/l)
Sham	21.85±3.96^a^	19.90±1.02^a^
I/R	46.77±6.54	35.20±3.34
PPC+I/R	33.54±4.95^a^	23.07±1.73^a^

Data are presented as the mean ± standard deviation; n=6.

a P<0.05 vs. I/R.

Sham, sham-operated animal without ligation; I/R, 30 min ischemia followed by 2 h reperfusion; PPC+I/R, administration of FCG for 5 days prior to the induction of myocardial ischemia. FCG, ferulic acid (300 mg/kg), cinnamic acid (200 mg/kg) and glycyrrhizic acid (50 mg/kg); TNFα, tumor necrosis factor-α; ICAM-1, intercellular adhesion molecule-1.

**Table VIII:** Effects of FCG pretreatment on serum IL-1β, IL-6 and CK-MB levels in the rat myocardium following ischemia-reperfusion.

Group	IL-1β (ng/l)	IL-6 (ng/l)	CK-MB (U/l)
Sham	18.29±5.32^a^	12.11±2.11^a^	229.45±21.08
I/R	26.44±8.24	34.32±6.50	1025.50±88.19
PPC+I/R	19.20±2.47^a^	16.77±4.22^a^	427.54±59.18

Data are presented as the mean ± standard deviation; n=6.

a P<0.05 vs. I/R.

Sham, sham-operated animal without ligation; I/R, 30 min ischemia followed by 2 h reperfusion; PPC+I/R, administration of FCG for 5 days prior to the induction of myocardial ischemia. FCG, ferulic acid (300 mg/kg), cinnamic acid (200 mg/kg) and glycyrrhizic acid (50 mg/kg); IL, interleukin; CK-MB, MB-isoenzyme of creatine kinase.

## Abbreviations

MDA: malondialdehyde
SOD: superoxide dismutase
TNFα: tumor necrosis factor-α
IL-1β: interleukin-1β
IL-6: interleukin-6
ICAM-1: intercellular adhesion molecule 1
PPARα: peroxisome proliferator-activated receptor α
I-κBα: inhibitory-κBα
NF-κB: nuclear factor-κB
CK-MB: MB-isoenzyme of creatine kinase
DGSN: Dang-gui-si-ni-tang
